# Dental service utilization and the COVID-19 pandemic, a micro-data analysis

**DOI:** 10.1186/s12903-023-03740-2

**Published:** 2024-01-04

**Authors:** Amir akbari, Mohammad Reza khami, Amine Beymouri, Solmaz Akbari

**Affiliations:** 1 Finance & Business Economics, DeGroote School of Business, McMaster University, Toronto, Canada; 2 Research Center for Caries Prevention, Dentistry Research Institute, Department of Community Oral Health, School of Dentistry, Tehran University of Medical Sciences, Tehran, Iran; 3 Department of Periodontics, School of Dentistry, Tehran University of Medical Sciences, Tehran, Iran

**Keywords:** Dental economics, Health service accessibility, Socioeconomic factors, Oral health utilization

## Abstract

**Background:**

Global crises and disease pandemics, such as COVID-19, negatively affect dental care utilization by several factors, such as infection anxiety, disrupted supply chains, economic contraction, and household income reduction. Exploring the pattern of this effect can help policy makers to be prepared for future crises. The present study aimed to investigate the financial impact of COVID‐19 disruptions on dental service utilization.

**Methods:**

Data on the number of dental services offered in Dental School Clinics of Tehran University of Medical Sciences was collected over a period of two years, before and after the initial COVID-19 outbreak in Iran. School of Dentistry operates two clinics; one with competitive service fees and one with subsidies. Regression analyses were performed to determine the effect of the pandemic on the number of dental services divided by dental treatment groups and these clinics. The analyses were adjusted for seasonal patterns and the capacity of the clinics.

**Results:**

There was a significant drop in dental services offered in both clinics across all dental groups in the post-COVID period (on average, 77 (39.44%) fewer services per day). The majority of the procedure loss happened in the Private clinic. Adjusting for seasonal patterns and the service capacity, regression results documented 54% and 12% service loss in Private and Subsidized clinics following the pandemic, respectively. Difference-in-difference analysis documented that the Subsidized clinic performed 40% more treatments than the Private clinic in the post-COVID period.

**Conclusions:**

Pandemic –reduction in dental care utilization could have long-term ramifications for the oral health of the population, and policymakers need to provide supportive packages to the affected segments of the economy to reverse this trend.

**Supplementary Information:**

The online version contains supplementary material available at 10.1186/s12903-023-03740-2.

## Background 

The COVID-19 pandemic, the most important global health issue in 2020–2021, seriously impacted the lifestyle and well-being of individuals worldwide [1]. It caused an economic catastrophe, led to widespread unemployment, and crippled the operations of many entities, including dental clinics [2, 3].

Due to the respiratory transmission and the nature of dentistry practices, dental clinics are high-risk locations for the spread of COVID-19 infection between patients and care providers [3, 4]. This negatively impacted the demand for dental care treatments; patients fearing the risk of contracting COVID-19 delayed or canceled their visits to dental clinics [5, 6]. In response, this sector of healthcare was forced to implement additional and costly protective measures, both in operating and post-operating procedures, in order to perform its routine tasks [3, 7, 8]. Furthermore, in the early phases of the pandemic, it had to limit operations to emergency dental treatments. Then gradually started performing elective dental treatments only after the abundance of personal protective equipment [9, 10] and widespread vaccination. In addition to shifts in the demand side and supply chain disruptions, frequent government-imposed lockdowns during the pandemic and service providers’ cross-infection anxiety have also been suggested as factors that challenged the access and utilization of dental services [11, 12]. However, the deterioration of dental care affordability, for instance, due to job loss, is often discussed as the main reason for lower dental service utilization in the later phases of the pandemic [2, 5, 13–16]. These will significantly negatively impact community health in the medium and long term, and it is even expected to have more long-term costs on healthcare, insurance, and household health costs [17].

The literature on the economic impact of COVID-19 in dentistry often relies on survey-based research or simulation modeling approaches to understand the underlying mechanism of supply and demand shifts for dental services [2, 10–12, 14, 18–20]. Survey studies indicated lower dental care demands during the COVID-19 pandemic in different parts of the world [13, 14, 20]. They reported several factors influencing dental care utilization, such as gender, age, education level, income level, geographic location, general health status, and dental insurance status [7, 16, 21]. As many of these factors are closely tied to household economic conditions, it is expected that dental care utilization would fluctuate, to some degree, with macroeconomic conditions [22–24]. For instance, Kotas and Dimitris (2017), based on the household budget survey in Greece, estimated that the household expenses for dental care decreased by about 57–59% during the Greek economic crisis between 2009 and 2014 [13]. An extensive literature on economic development documented that household disposable income decreased during the COVID-19 pandemic in countries around the world [25, 26]. Based on the World Bank’s reports, the household disposable income per household in Iran was 8.00, 6.23, 8.66, and 10.86 thousand US dollars for 2019, 2020, 2021, and 2022 respectively. The drop in this value for the COVID-19 pandemic suggested that the purchasing power of Iranian households decreased.

Dental health service use is also significantly associated with health insurance status, which is commonly offered by employers and is weaker during large economic downturns [14, 18–20, 26, 27]. The survey studies also reported substantial drops in dental care provision as well as a reduction in patient referrals during the COVID-19 pandemic [6, 11, 17]. Multiple simulation-based studies showed that economic contraction during the COVID-19 pandemic negatively impacted dental care utilization and overall dental health [2, 16–18].

As discussed above, policymakers referred to surveys and simulation analysis to design effective social transfers and subsidies. However, these reports and analyses suffer from common limitations. Often responding participants to surveys are not entirely randomly chosen, and the selection bias concerns persist. Simulation-based analyses are also not free of biases; they suffer from over-simplification and arbitrary model assumptions. Thus, we designed our study to complement the above research and to provide further evidence on the demand-based channel for dental service utilization in the post-pandemic period.

To this aim, we chose to study fluctuation in dental services utilization resulting from the COVID-19 pandemic in Dental School Clinics of Tehran University of Medical Sciences (TUMS). School of Dentistry operates two clinics, one with subsidy (labeled as S-Subsidized), where under- and postgraduate students perform dental procedures under the direct supervision of faculty members. The other clinic (labeled as P-Private) is operated by (junior) faculty members in the same location during the evenings, and their services are not financially subsidized. Both clinics offer the same range of services in all major treatment modalities. The discount for the procedures in the Subsidized clinic is 35% to 55%, depending on the treatment, compared to the same service in the Private clinic. By studying differential services offered by these clinics, we control for various socio-economic factors that affect service utilization and thus better evaluate supply and demand channels for dental services.

## Methods

The present study is an observational, cross-sectional study of dental services in the School of Dentistry, TUMS clinics. The study protocol was approved by the Ethical Committee of the TUMS (ethical code: IR.TUMS.DENTISTRY.REC.1401.106). The patients’ dental service data were collected from the school’s health information system (HIS) database after their anonymity grace period in accordance with relevant guidelines of TUMS. We collected the anonymized, micro-level data of the services offered to patients in clinics of the School of Dentistry, TUMS, over a period of two years, before and after the initial COVID-19 outbreak in Iran. All methods carried out in the study were performed with the relevant guidelines and regulations.

**Fig. 1:**
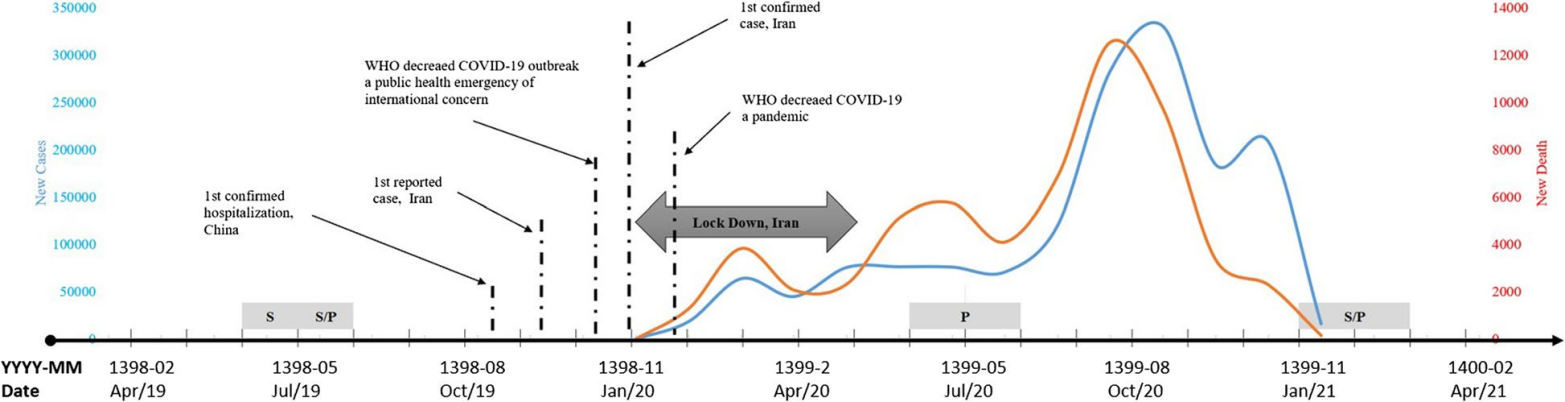
The operating dates of Tehran University’s dental clinics. The x-axis shows the time in year-month for the events in Iranian (YYYY-MM) and Christian (MMM-YY) calendars. The gray boxes highlight the periods that Subsidized (S) and Private (P) clinics were not functioning at full capacity due to University’s calendar. The COVID-19 lockdown period is highlighted with a gray arrow. The number of new COVID-19 cases and deaths are shown with blue and red lines on the left and right y-axes, respectively. These lines depict the major COVID-19 waves in Iran (Source: World Health Organization and Iran’s Ministry of Health and Medical Education)

In a typical academic year, dental treatment services are actively provided for eight months in the School of Dentistry. University’s academic calendar and statutory holidays determine the clinics’ operating dates. Following the official announcement of the COVID-19 outbreak in Iran on February 22, 2020, the School of Dentistry suspended all routine services and limited its procedures to emergency care from February 24, 2020, to June 8, 2020. Since then, the school and relative clinics have been closed twice more, once for four weeks and once for three weeks.^1^ Figure 1 describes the timeline of the operating dates for these clinics.

Following these, we considered the initial announcement of the COVID-19 outbreak in Iran as a reference date for the beginning of the pandemic; thus, April 21, 2019, to February 24, 2020, was considered the pre-COVID period, and June 8, 2020, to April 21, 2021, as the post-COVID period. The number of all dental treatments carried out was collected from HIS database per day and by dental treatment groups (orthodontic treatments, prosthodontic therapies, periodontal treatments, restorative therapies, pediatric treatments, and root canal treatments). These were categorized separately based on the Subsidized and Private clinics. In the baseline analysis, only the days with at least one service in either of the clinics were included. In the difference-in-difference analysis, the days were further excluded if each dental department was not operating in both clinics. List of some of dental services offered in each dental groups as well as the relative treatment price between the two clinics were listed in Supplementary file 1.

### Statistical analysis

The mean number of dental services in each dental treatment group, before and after the COVID-19 outbreak, was separately calculated in each clinic. The relationship between the number of daily dental services and a COVID indicator event (the days after June 8, 2020, took a value of 1, and the rest 0) was studied through regression analysis (Time series Multivariate Linear Regressions through Even Study specification). Seasonal trends in the demand for dental services, rooted in national and religious events, or the university’s calendar events shaped dental service utilization in our sample, So the regressions also included the month-of-the-year fixed effects, which captured seasonal trends in patient flows. All the statistical analyses were carried out using the microlevel data, at daily frequency, and disaggregated at the clinic-level (S and P), and dental treatment group (Prosthodontics, Periodontics, Pediatric, Orthodontic, Endodontic, and Restorative). This allows us to present robust statistical inferences from the underlying mechanism. The estimated slope coefficient for the COVID indicator showed how many fewer (or more) treatments were offered after the COVID-19 outbreak in the clinics. The absolute drop or rise in the number of dental services, ignoring the size of each clinic and dental group, could be misleading in determining which category was impacted the most. In response, the analysis was expanded by adjusting for the service capacity. Service capacity identifies the processing ability of each clinic and dental group, as in the clinics of the Faculty of Dentistry, the number of services that each group offers varies. For a fair comparison between groups, during the COVID-19 pandemic, we scale the number of daily services offered in each group and clinic by their respective service capacity.

Lastly, a difference-in-difference statistical analysis was performed to control further external factors. This distinguished how much fewer services the Private clinic offered compared to the Subsidized clinic. The dependent variable was the volume of services each dental group offered daily in the S-clinic minus that of the P-clinic. The independent variables were the COVID indicator and the seasonal trends in the data. The data were analyzed using the statistical software R, based on its Testing Linear Regression Models (LMTEST) and Linear Mixed-Effects Models (LME4) libraries at *p* < 0.05 significant level. The full set of estimated results from these regressions was included in Supplementary file 2, where the regression formula for each analysis were included too.

## Results

Throughout our sample, the Subsidized and Private clinic operated for 354 days, pre-COVID and during COVID era, (157 + 197), and 336 days (180 + 156), respectively. Table 1 tabulates the summary statistics of the number of services in each clinic and each dental group. A small heterogeneity was also observed in the number of days the dental groups operated in each period. Overall, the operating capacity and patients’ demand for these groups differ in the cross-section.

**Table 1:** Summary of the service volume in each clinic, Subsidized (S), Private (P), and together (S + P), and per dental group

Group	Clinic	#DAYS		TOTAL		MEAN	
		pre-COVID	post-COVID	pre-COVID	post-COVID	pre-COVID	post-COVID
Prosthodontics	S	142	164	1,340	1,209	9.44	7.37
	P	141	85	835	293	5.92	3.45
	S + P	283	249	2,175	1,502	7.69	6.03
Periodontics	S	149	186	1,462	1,840	9.81	9.89
	P	172	109	1,941	547	11.28	5.02
	S + P	321	295	3,403	2,387	10.60	8.09
Pediatrics	S	157	193	6,116	4,894	38.96	25.36
	P	179	119	7,008	2,807	39.15	23.59
	S + P	336	312	13,124	7,701	39.06	24.68
Orthodontics	S	106	157	319	932	3.01	5.94
	P	174	110	1,387	580	7.97	5.27
	S + P	280	267	1,706	1,706	6.09	5.66
Endodontic	S	142	184	907	810	6.39	4.4
	P	176	148	3,146	1,169	17.88	7.9
	S + P	318	332	4,053	1,979	12.75	5.96
Restorative	S	153	185	1,861	1,852	12.16	10.01
	P	179	146	6,040	2,666	33.74	18.26
	S + P	332	331	7,901	4,518	23.80	13.65
All Groups	S	157	197	12,005	11,537	76.46	58.56
	P	180	156	20,357	8,062	113.09	51.68
	S + P	337	353	32,362	19,599	96.03	55.52

The statistics are presented for pre-and post-COVID subperiods. Column #DAYS shows the number of days each group performed at least one treatment in each clinic. Column MEAN presents the average number of services offered in each category on each day

**Fig. 2:**
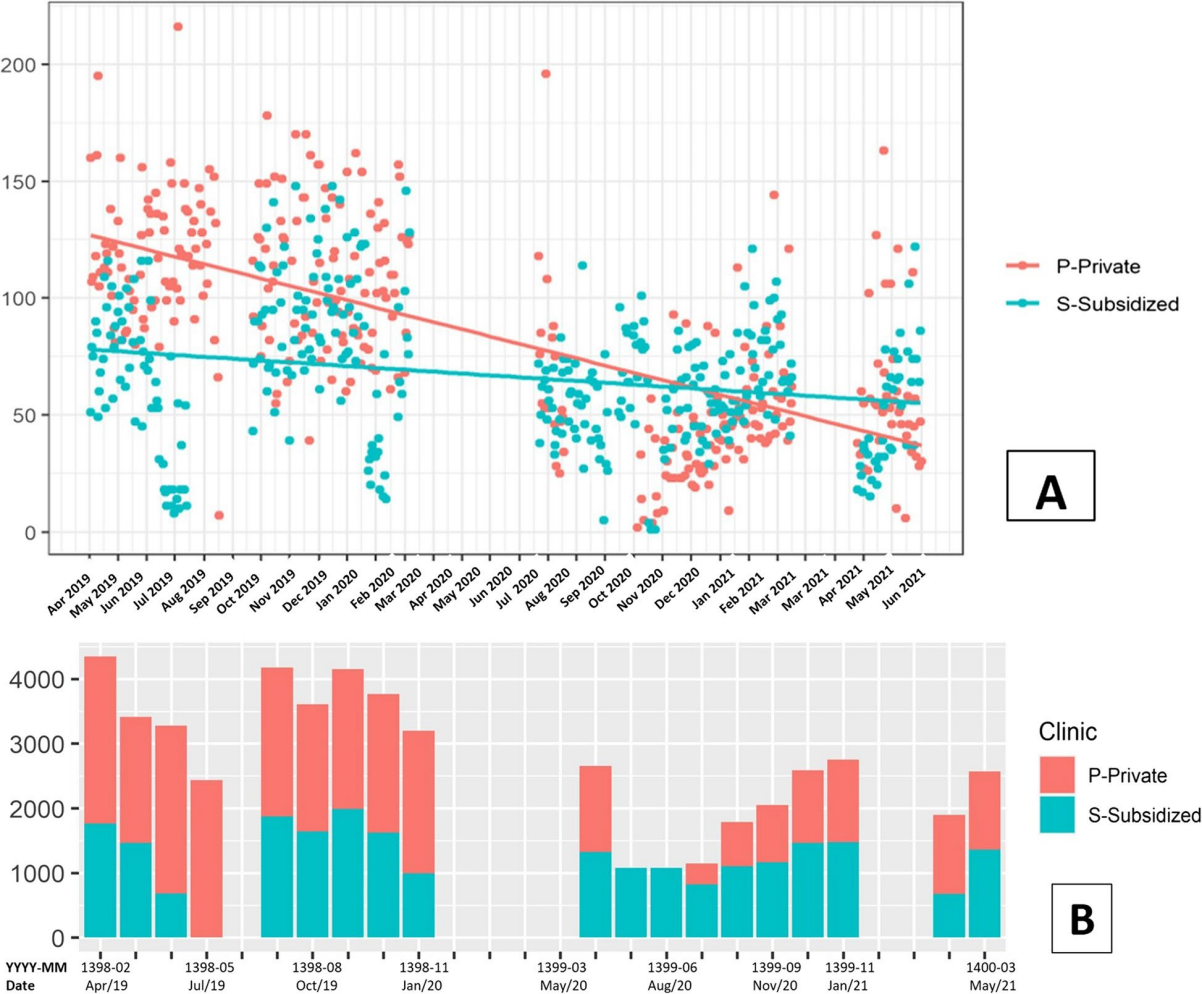
The total number of dental services at the daily frequency (**A**) and aggregated in a month (**B**). The height of each bar indicates the number of services in the Subsidized (S) and Private (P) clinics. The x-axis shows the time in year-month for the events in the Iranian (YYYY-MM) calendar and corresponding AD calendar. The data ranges from April 21, 2019, to April 21, 2021

Figure 2 visualizes the total number of treatments offered by all dental groups in Subsidized and Private clinics, at the daily frequency (Fig. 2A) and aggregated in a month (Fig. 2B). First, there were variations in the number of services over time, even in the normal period, which is consistent with the seasonality in demand for dental services. Second, the volume of dental services dropped significantly following the pandemic; on average, in a month, clinics offered 3,596 and 1,960 services in the pre- and post-COVID periods, respectively, equivalent to 45.49% fewer services in a month after pandemic.

Excluding 1399–04 (June 2020), a gradual increase in the number of services was observed in the post-COVID period, consistent with improved protection measures and reduced infection anxiety in the population after the first wave of COVID-19. The large number of recorded services in 1399–04 month was most likely related to the backlog of patients who could not receive the much-needed dental treatments during the lockdown period. Table 2 presents the analysis for the differences in number of daily dental services in the pre- and post-COVID period by each dental group and clinics. There was a significant drop in the number of dental services offered in both clinics across all dental groups, as documented by the negative slope coefficients of the clinics and dental group regressions (Table 2, Panel A. Adjusted for seasonal effects, there were, on average, 77 fewer services per day (60 and 17 fewer services in P-clinic and S-clinic, respectively) in the post-COVID period.

**Table 2:** Regressions of daily dental service and the COVID indicator. Panel A presents the estimated slope coefficients for the number of daily services, and Panel B presents the percentage changes in the number of daily services in the post-COVID period after adjusted by service capacity for each clinic and per dental group

Clinic	Prosthodontics	Periodontics	Pediatric	Orthodontic	Endodontic	Restorative	All Groups
Panel A							
S + P	-4.45^a^	-6.34^a^	-32.51^a^	-2.45^a^	-12.06^a^	-19.61^a^	-77.43^a^
S	-2.29^a^	0.67	-14.34^a^	2.33^a^	-1.64^a^	-2.29^a^	-17.55^a^
P	-2.84^a^	-7.35^a^	-20.63^a^	-3.82^a^	-9.54^a^	-16.80^a^	-60.97^a^
Panel B							
S + P	-% 23^a^	-% 27^a^	-% 43^a^	-% 7	-% 48^a^	-% 40^a^	-% 41^a^
S	-% 6	% 27^a^	-% 29^a^	% 87^a^	-% 9	-% 1	-% 12^b^
P	-% 37^a^	-% 55^a^	-% 39^a^	-% 30^a^	-% 54^a^	-% 46^a^	-% 54^a^

S and P denote the Subsidized and Private clinics, respectively, whereas S + P denotes the total services offered by both clinics. The regressions also include the month-fixed effects

^a^ and ^b^  denote statistical significance at the 1% and 5% *p*-value levels, respectively

Table 2, Panel B presents the percentage changes in the number of daily services in the post-COVID period, which the dependent variables were scaled by the service capacity (i.e., size) of each dental group in each clinic. This analysis, maybe more clearly, documented that the drop in the number of services offered in the post-COVID period was initiated from the Private clinic; all dental groups in P-clinic had fewer services, and their drop was much larger (and statistically significant) than the corresponding drop in the Subsidized clinic. These changes were relatively small or statistically insignificant for the dental groups in the S-clinic.

A difference-in-difference analysis was performed to clarify the differences between the demand for dental services from Subsidized and Private clinics. Table 3 shows the number and percentage of excess treatments performed in the S-clinic compared to the P-clinic. The term S-P in the table denotes the number of services offered by clinic S minus that of clinic P. In the pre-COVID period, the P-clinic offered significantly more dental services than the S-clinic, with up to 933 more services per month. However, this trend flipped after the COVID-19 outbreak, when the P-clinic offered up to 418 fewer services than the S-clinic (Table 3, Panel A). Adjusting for the capacity of each group, the results in Panel B further ensure that the P-clinic offered much fewer services in the post-COVID period compared to the S-clinic. Analyzing dental groups separately, the largest drop in the number of treatments was observed for the restorative group. However, the largest drop (based on service capacity) was observed for the orthodontics and then for the periodontics groups in P-clinic after COVID-19 outbreak. The pediatric group was the least affected group in our sample, where the estimate had no statistical significance.

**Table 3:** Regressions of relative dental services of the S-clinic in excess of the P-clinic and COVID indicator. Panels A and B present the estimated slope coefficients from regressions of the number of and percentage of daily changes in service, respectively, in the post-COVID period for each clinic and per dental group^b^

	Prosthodontics	Periodontics	Pediatric	Orthodontic	Endodontic	Restorative	All Groups
Panel A							
S-P	0.51	7.09^a^	2.19	4.84^a^	7.35^a^	12.65^a^	41.68^a^
# Days	180	248	273	191	273	282	304
Panel B							
S-P	% 33^c^	% 91^a^	% 13	% 109^a^	% 43^a^	% 40^a^	% 40^a^

Row #DAYS presents the number of working days where a specific dental group operated in both clinics. The regressions also included the month-fixed effects. The results are presented separately for each dental group at the daily frequency

^a, ^^b ^and ^c^ denote statistical significance at the 1%, 5%, and 10% *p*-value levels, respectively

## Discussion 

The current exploratory research aimed to explore and determine the shift in dental service utilization patterns depending on the overall economic impact of COVID-19. For this purpose, we exploited the micro-level data of dental treatments that the Subsidized and Private clinics of Tehran University offered in a span of two years around the initial COVID-19 outbreak in Iran.

First, a significant reduction in the volume of treatments was documented in these clinics; they, in total, offered 32,362 services across all dental groups in the pre-COVID period but only 19,599 in the post-COVID period, a staggering 39.44% drop at the daily frequency. This pattern was observed in all dental treatment groups, experiencing 30% to 51% drops, except for the orthodontics group in the S-clinic. A significant decline was observed in daily number of endodontic treatments. The shorter length of treatment and lower expenses might have pushed the patients to extract their painful teeth instead of saving them [28]. A similar shift in demand for dental services was also observed during the economic crisis in Greece [13]. Other considerations, such as parents’ extra concern about children’s health [29], seemed to be the primary explainer of the 41% reduction in pediatric treatments. In the same way, an extensive decline in children’s oral health status and dental care utilization was reported during the COVID-19 pandemic in 2020 with one year earlier [30, 31]. Our findings were in accordance with some studies, showed that COVID-19 lockdown regulations reduced dental service utilization, particularly preventive, periodontic, and prosthetic treatments [18, 32]. In addition, centers with higher tariffs and lower insurance coverage experienced more revenue declines [19]. Exploiting a similar approach, Lo Nigro et al. found that the additional protective measures introduced after the pandemic resulted in a significant profitability loss and dental service utilization [20]. Consistent with the evidence in Villarim et al. [6] on the increased cost of dental visits and consultations, these analyses conclude that the COVID-19 pandemic imposed a significant negative financial burden on the dental economy and suggest a more proactive stance is necessary to support dental care provision under pandemic constraints [33].

Overall, the literature has suggested several potentially influential factors for patient and procedure volume loss during the pandemic. First, the unwillingness of patients due to infection anxiety [6, 7]. Second, clinics were initially restricted to offering only emergency care [34]. Third, the pandemic disrupted the global supply chain, reducing access to certain products, including preventive equipment and supplies. This further restricted the service capacity of dental clinics. Lastly, and maybe more importantly, the pandemic led to a severe economic recession and widespread job losses. It imposed significant financial concerns on large population segments, forcing them to seriously reprioritize their disposable income and update their precautionary saving for essential needs. The findings of Choi et al. [7, 14] suggest that as a result of these forces, a portion of patients were pressed to reduce or shift their demand for dental care, resulting in deterioration of the oral health of the population. Worsened socioeconomic condition and household income reduction during COVID-19 pandemic were also found to be associated with dental pain and deteriorated oral health [35].

Beside these abovementioned factors, other factors such as, the quality of the services, their ease of access, availability of specialized services, or their affordability can potentially drive the supply and demand for dental services in our sample. However, establishing the key drivers and their marginal importance is empirically challenging. Some of these factors are not easily measurable or observable, the data for some is not freely accessible in our sample, and some of these factors have reinforcing effects. To establish that the financial channel is a key driver, a novel empirical approach was implemented to control for a series of these factors. We exploited the unique setting at the Faculty of Dentistry, which manages two comparable dental clinics (S and P), which are equipped with similar dental facilities, offer similar services, are located in the same place, follow the policies of the same institution, Tehran University, but charge different service fees. If any of the abovementioned factors affected patients of clinics S and P simultaneously in the post-COVID period, the spread between the number of services clinics S and P will stay unchanged; both clinics as a result of that clinic will offer fewer services. Our study builds on this intuition and shows that the financial channel, the affordability of dental services for patients, was an important factor for the observed decrease. The Difference-in-Difference methodology allowed us to isolate the financial channel, controlling for factors that are commonly driving the supply and demand for dental services.

We argued that this financial incentive is the primary driver of the 46 fewer daily treatments that were performed in the P-clinic versus the S-clinic. Difference-in-difference analysis documented that the S-clinic performed 40% more treatments than the P-clinic in the post-COVID period. Comparing the dental groups, the most prominent effects are observed in the orthodontics and periodontics groups, which involves the most expensive dental care and surgical procedures; these groups in the S-clinic performed 109% and 91% more treatments in the post-COVID period than the same group in the P-clinic. Interestingly, the Pediatric group is the least affected group for which we did not document a meaningful difference between the Private and the Subsidized clinics’ treatment volume. This is consistent with the notation that the demand for Pediatric treatments has small price elasticity, possibly because parents might delay or cancel their children’s dental care visits less so in response to higher perceived expenses. To conclude, our findings are suggestive that economic contractions and the consequent inflation and high daily expenses explain the large drop in patient volume in the dental care sector.

Similar changes in patient volumes and dental care use have been reported in other countries and other economic crises [7, 13, 18, 36, 37]. Increases in the number of tooth extractions and decreases in prosthetic treatments, which had a high cost to the patients, have been reported in a study evaluating the effects of the Greek economic crisis [13]. A similar pattern was observed in the US during the 2008–09 housing market crash when the uninsured segment of the population noticeably lost their access to dental care services [38]. This economic meltdown spread internationally with similar consequences. For instance, in Spain, the number of unmet dental care requirements increased from 2007 to 2011, especially among the less affluent people, due to large unemployment in this period [39].

The Subsidized and Private clinics of TUMS could provide a testable framework for policymaking during large disruptions such as the COVID-19 pandemic, particularly in less affluent populations or those with less widespread dental insurance programs. It is well documented that dental insurance coverage and house hold income strongly influence the demand for dental care [2, 31]. Following the COVID-19 pandemic, dental care use has been rebounded slower in the publicly insured population compared to privately insured patients [7] and patients received more invasive dental procedures due to delayed treatment [40]. Generalizing our findings, we argued that offering a 35% to 55% subsidy could alleviate the negative financial burdens of pandemics, especially for the more expensive dental treatments up to 109%. Due to privacy reasons the retrieval of the details of the type of treatment and their frequency for each patient was not feasible and this could be considered as a drawback of our study. We acknowledge that the evaluation of such information could have provided more precise insight on trends in dental care utilization during pandemic shutdown periods. During the COVID era, especially at the beginning of the pandemic, some changes enforced in the dental student’s curriculum and decreases in the number of requirements introduced by the dental schools in order to protect students from contamination. A similar restriction, with a similar goal to protect dentists and dental staff, was imposed in the Private clinic. This policy reduced the supply of dental treatments in both clinics in somehow similar way. The difference in difference methodology were implemented to control such factors, however, this could be a source of possible bias in the evaluation of the changes in the care provision especially in the S-clinic.

## Conclusion

Subsidizing dental treatments for the less affluent segments of the population, for instance, in the form of social health insurance, could help with inequality in access to oral health care [3]. In the presence of a risk of developing new infectious diseases due to climate change and population growth [20], we claim that our findings can have direct implications for potential future disruptions that might occur as a result of epidemics and pandemics similar to COVID-19. Therefore, our findings empower policymakers in designing more inclusive dental care for all socioeconomic groups of the population.

### Supplementary Information

**Additional file 1: Supplementary Table 1.** List of dental services that are offered in each dental group in TUMS, as well as the subsidy patients receive in Clinic S, in percentages, compared to Clinic P.

**Additional file 2: Fig. A1.** Service Capacity. **Fig. A2.** Treatments by Clinics. **Table A1.** Level of Dental Services and the Pandemic (S+P). **Table A2.** Level of Dental Services and the Pandemic (S). **Table A3.** Level of Dental Services and the Pandemic (P). **Table A4.** Level of Dental Services and the Pandemic (S&P). **Table A5.** Level of Dental Services and the Pandemic (DID test). **Table A6.** Change in Dental Services and the Pandemic (S+P). **Table A7.** Change in Dental Services and the Pandemic (S). **Table A8.** Change in Dental Services and the Pandemic (P). **Table A9.** Change in Dental Services and the Pandemic (S&P). **Table A10.** Change in Dental Services and the Pandemic (DID test). **Table A11.** Level of Relative Dental Services and the Pandemic. **Table A12.** Level of Relative Dental Services and the Pandemic.

## Data Availability

The datasets used and/or analyzed during the current study are available from the corresponding author on reasonable request.

## Abbreviations

TUMS: Tehran University of Medical Sciences
HIS: Health information system
S-clinic: Subsidized clinic
P-clinic: Private clinic

## Footnotes

1 After the COVID-19 outbreak, TUMS’s clinics were the only operational university-run center in the city for several months, where all dental services, selective and elective ones, were provided in both subsidized and private clinics.

## References

[1] Jiang Y, Tang T, Mei L, Li H (2022). COVID-19 affected patients’ utilization of dental care service. Oral Dis..

[2] Nasseh K, Vujicic M (2020). Modeling the impact of COVID-19 on US dental spending. Health policy institute research brief American dental association.

[3] Ali S, Farooq I, Abdelsalam M, AlHumaid J (2020). Current clinical dental practice guidelines and the financial impact of COVID-19 on dental care providers. Eur J Dent..

[4] Izzetti R, Nisi M, Gabriele M, Graziani F (2020). COVID-19 transmission in dental practice: brief review of preventive measures in Italy. J Dent Res.

[5] Villarim NLdS, Muniz IdAF, Perez DEdC, Martelli Junior H, Machado RA, Cavalcanti YW (2022). Evaluation of the economic impact of COVID-19 on Brazilian private dental clinics: A cross-sectional study. Work..

[6] Guo H, Zhou Y, Liu X, Tan J (2020). The impact of the COVID-19 epidemic on the utilization of emergency dental services. J Dent Sci.

[7] Choi SE, Simon L, Basu S, Barrow JR (2021). Changes in dental care use patterns due to COVID-19 among insured patients in the United States. J Am Dent Assoc..

[8] Meethil A, Saraswat S, Chaudhary P, Dabdoub S, Kumar P (2021). Sources of SARS-CoV-2 and other microorganisms in dental aerosols. J Dent Res.

[9] Ren Y, Feng C, Rasubala L, Malmstrom H, Eliav E (2020). Risk for dental healthcare professionals during the COVID-19 global pandemic: An evidence-based assessment. J Dent.

[10] Campus G, Betancourt MD, Cagetti MG, Giacaman RA, Manton DJ, Douglas GV (2021). The COVID-19 pandemic and its global effects on dental practice. An international survey. J Dent.

[11] Mahdee AF, Gul SS, Abdulkareem AA, Qasim SSB (2020). Anxiety, practice modification, and economic impact among Iraqi dentists during the COVID-19 outbreak. Front Med.

[12] Consolo U, Bellini P, Bencivenni D, Iani C, Checchi V (2020). Epidemiological aspects and psychological reactions to COVID-19 of dental practitioners in the Northern Italy districts of Modena and Reggio Emilia. Int J Environ Res Public Health.

[13] Kostas D, Dimitris N (2017). The effects of economic crisis on the demand and supply of the dental services in Greece. J Int Soc Prevent Commun Dent.

[14] Choi S, Simon L, Riedy C, Barrow J (2021). Modeling the impact of COVID-19 on dental insurance coverage and utilization. J Dent Res.

[15] Guay AH, Blatz A (2019). The effect of the Great Recession on the demand for general oral health care and orthodontic care. J Am Dent Assoc.

[16] Patel N (2020). Impact on dental economics and dental healthcare utilization in COVID-19: an exploratory study. J Adv Oral Res.

[17] Abdulkareem AA, Abdulbaqi HR, Alshami ML, Al-Rawi NH (2021). Oral health awareness, attitude towards dental treatment, fear of infection and economic impact during COVID-19 pandemic in the Middle East. Int J Dental Hygiene.

[18] Schwendicke F, Krois J, Gomez J (2020). Impact of SARS-CoV2 (Covid-19) on dental practices: Economic analysis. J Dent.

[19] Lo Nigro G, Bizzoca ME, Lo Muzio L, Campisi G (2020). The management of dental practices in the post-COVID 19 era: an economic and operational perspective. Int J Environ Res Public Health.

[20] Alarcón MA, Sanz-Sánchez I, Shibli JA, Treviño Santos A, Caram S, Lanis A (2021). Delphi Project on the trends in Implant Dentistry in the COVID-19 era: Perspectives from Latin America. Clin Oral Implant Res.

[21] Scherrer C, Naavaal S, Lin M, Griffin S (2022). COVID-19 Pandemic Impact on US Childhood Caries and Potential Mitigation. J Dent Res.

[22] Wall TP, Vujicic M, Nasseh K (2012). Recent trends in the utilization of dental care in the United States. J Dent Educ.

[23] Michener J, Brower MT (2020). What’s policy got to do with it? Race, gender & economic inequality in the United States. Daedalus..

[24] Whitman A, De Lew N, Chappel A, Aysola V, Zuckerman R, Sommers BD (2022). Addressing social determinants of health: Examples of successful evidence-based strategies and current federal efforts. Off Heal Policy.

[25] Dauderstädt M (2022). International inequality and the COVID-19 pandemic. Intereconomics.

[26] Blundell R, Costa Dias M, Cribb J, Joyce R, Waters T, Wernham T, Xu X (2022). Inequality and the COVID-19 Crisis in the United Kingdom. Ann Rev Econ.

[27] Maupome G, Scully AC, Yepes JF, Eckert GJ, Downey T (2022). Trends in dental insurance claims in the United States before and during the SARS-CoV-2 pandemic in 2020. J Public Health Dent.

[28] Darestani MN, Akbari A, Yaghobee S, Taheri M, Akbari S (2022). COVID-19 Pandemic and Periodontal Practice: The Immunological, Clinical, and Economic Points of View. Biomed Res Int..

[29] Malhão EC, de Almeida GF, Ferreira CM, Lima DL, Casarin M, Pappen FG (2021). Endodontic treatment during COVID-19 pandemic: Economic perception of dental professionals. Bras J Oral Sci.

[30] Farsi D, Farsi N (2021). Mothers’ Knowledge, Attitudes, and Fears About Dental Visits During the COVID-19 Pandemic: A Cross-sectional Study. J Int Soc Prev Community Dent..

[31] Karande S, Chong GTF, Megally H (2023). Changes in dental and medical visits before and during the COVID-19 pandemic among U.S. children aged 1–17 years. Community Dent Oral Epidemiol..

[32] Lyu W, Wehby GL (2022). Effects of the COVID-19 pandemic on children’s oral health and oral health care use. J Am Dent Assoc.

[33] Watt RG (2020). COVID-19 is an opportunity for reform in dentistry. The Lancet.

[34] Beauquis J, Petit A-E, Michaux V, Sagué V, Henrard S, Leprince J (2021). Dental emergencies management in COVID-19 pandemic peak: a cohort study. J Dent Res.

[35] Matsuyama Y, Aida J, Takeuchi K, Koyama S, Tabuchi T (2021). Dental pain and worsened socioeconomic conditions due to the COVID-19 pandemic. J Dent Res..

[36] Chamorro-Petronacci C, Martin Carreras-Presas C, Sanz-Marchena A, A Rodríguez-Fernández M, Maria Suarez-Quintanilla J, Rivas-Mundiña B (2020). Assessment of the economic and health-care impact of COVID-19 (SARS-CoV-2) on public and private dental surgeries in Spain: A pilot study. Int J Environ Res Public Health..

[37] Rector JM, Scully AC, Yepes JF, Jones JE, Eckert G, Downey T (2023). Financial Impact of COVID-19 on Dental Care for Pediatric Patients: a Dental Claims Review. Pediatr Dent.

[38] Kenney GM, McMorrow S, Zuckerman S, Goin DE (2012). A decade of health care access declines for adults holds implications for changes in the Affordable Care Act. Health Aff.

[39] Fernández SC, Ajuria AF, Martín JJ, Murphy MJ (2015). The impact of the economic crisis on unmet dental care needs in Spain. J Epidemiol Community Health.

[40] Leake J, Birch S (2008). Public policy and the market for dental services. Commun Dent Oral Epidemiol.

