# Supplementary material for: Dental service utilization and the COVID-19 pandemic, a micro-data analysis
Source: BMC Oral Health. 2024 Jan 4;24:16. doi: 10.1186/s12903-023-03740-2 (PMC10768144; doi:10.1186/s12903-023-03740-2)
Supplement: Supplementary file 1 — Additional file 1: Supplementary Table 1. List of dental services that are offered in each dental group in TUMS, as well as the subsidy patients receive in Clinic S, in percentages, compared to Clinic P. [file 12903_2023_3740_MOESM1_ESM.docx]

Supplementary table-1. List of dental services that are offered in each dental group in TUMS, as well as the subsidy patients receive in Clinic S, in percentages, compared to Clinic P.

| Dental Group | Example of Services they offer | Subsidy S/P |
| --- | --- | --- |
| Periodontics | Scaling/root planning  All types of periodontal flaps  Pre prosthetic surgeries  Soft and hard tissue augmentation, etc. | 35%  55%  55%  Depends to the type of procedure 55-120% |
| Prosthodontic treatments | Post and crown  Fixed prosthesis  Removable complete / and partial dentures  Resin bonded restorations, etc. | 55%  55%  60%  50% |
| Endodontic treatments | One, two and three root endodontic treatments  Re treatment root canal treatments  Apico surgeries, etc. | 40%  50%  - |
| Orthodontic therapies | Fixed orthodontic treatments  Removable orthodontic treatments, etc. | 55%  30% |
| Restorative therapies | All types of filling  Composite build-ups  Composite veneers, etc | 20-35%  30- 40%  55% |
| Pediatric treatments | All types of fillings on deciduous teeth  Extractions  Fissure sealant therapies  SS crowns  Preventive interventions  Minor orthodontic therapies in mixed dentition, etc. | 35%  40%  40%  55%  30%  55% |
