# Supplementary material for: Dental service utilization and the COVID-19 pandemic, a micro-data analysis
Source: BMC Oral Health. 2024 Jan 4;24:16. doi: 10.1186/s12903-023-03740-2 (PMC10768144; doi:10.1186/s12903-023-03740-2)
Supplement: Supplementary file 2 — Additional file 2: Fig. A1. Service Capacity. Fig. A2. Treatments by Clinics. Table A1. Level of Dental Services and the Pandemic (S+P). Table A2. Level of Dental Services and the Pandemic (S). Table A3. Level of Dental Services and the Pandemic (P). Table A4. Level of Dental Services and the Pandemic (S&P). Table A5. Level of Dental Services and the Pandemic (DID test). Table A6. Change in Dental Services and the Pandemic (S+P). Table A7. Change in Dental Services and the Pandemic (S). Table A8. Change in Dental Services and the Pandemic (P). Table A9. Change in Dental Services and the Pandemic (S&P). Table A10. Change in Dental Services and the Pandemic (DID test). Table A11. Level of Relative Dental Services and the Pandemic. Table A12. Level of Relative Dental Services and the Pandemic. [file 12903_2023_3740_MOESM2_ESM.pdf]

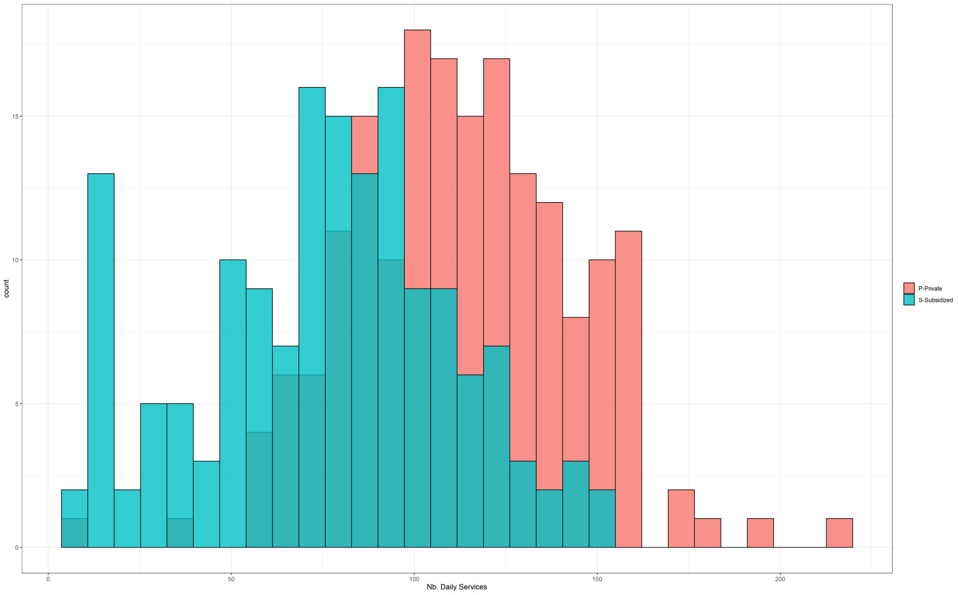

(a) pre-COVID

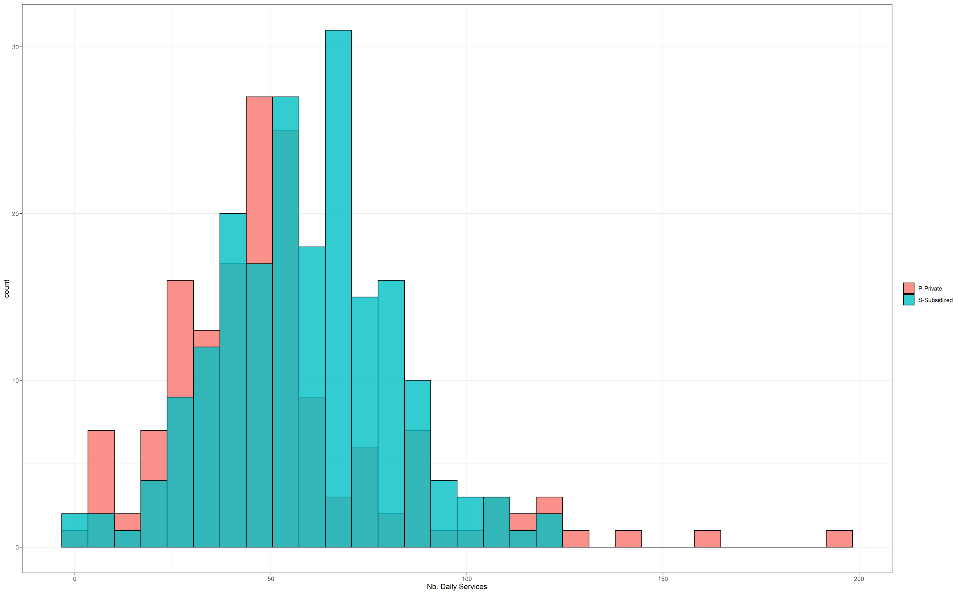

(b) post-COVID

Fig. A1. **Service Capacity.** The graph shows the histogram of the daily dental services offered in Subsidized (S) and Private (P) clinics of Tehran University of Medical Sciences by all treatment groups. The top and bottom panels present the results for the pre-COVID and post-COVID periods, respectively. The data ranges from April 21, 2019 to April, 21, 2021.

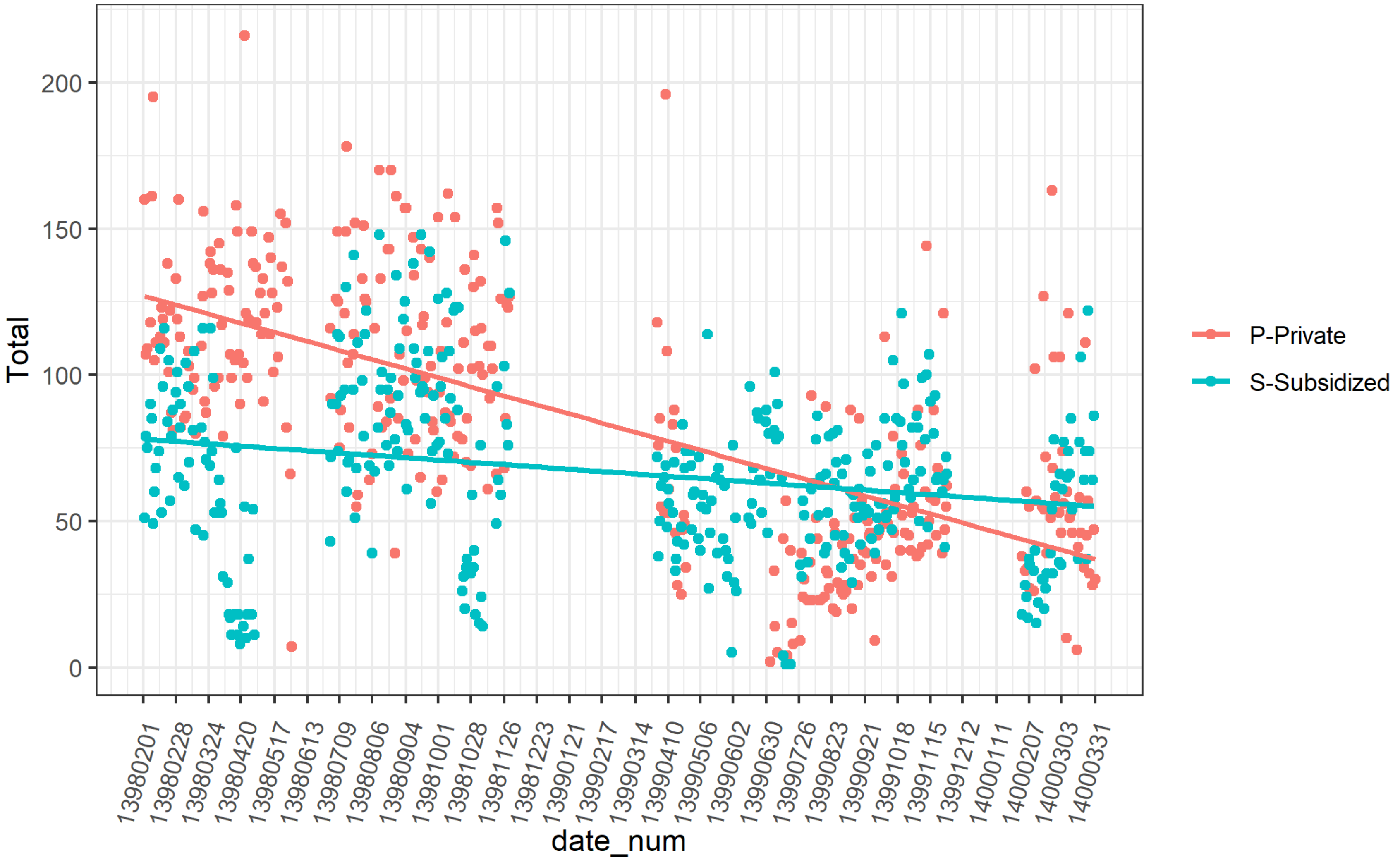

Fig. A2. **Treatments by Clinics.** The graph shows the total number dental treatments offered in Subsidized (S) and Private (P) clinics of Tehran University of Medical Sciences, at the daily frequency. In addition, the fitted trend line for each group is shown. The data ranges from April 21, 2019 to April, 21, 2021.

Table A1: Level of Dental Services and the Pandemic (S+P). The table presents the estimated slope coefficients for the number services offered in Dental School clinics of Tehran University of Medical Sciences by each dental treatment group and the pandemic indicator variable (COVID). The regressions also include the month-fixed effects. The sample period is from April 21, 2019, to April 21, 2021 at the daily frequency. P-values are estimated using robust standard errors (reported in parentheses). ***, **, and * denote statistical significance at the 1%, 5%, and 10% p-value levels, respectively.

$$\text{Service}_t^{\text{Group}_j} = \alpha + \beta^{\text{Group}_j}\,\text{COVID}_t + \gamma^m\,\text{Month}_t^m + e_t^{\text{Group}_j}$$

	Prosthodontics	Periodontics	Pediatric	Orthodontic	Endodontic	Restorative	All Groups
COVID	−4.45***	−6.34***	−32.51***	−2.45***	−12.06***	−19.61***	−77.43***
	(0.80)	(0.75)	(2.96)	(0.62)	(0.71)	(1.36)	(4.75)
Month_3	0.52	0.98	0.10	2.66**	1.03	−1.54	3.75
	(1.67)	(1.58)	(6.23)	(1.31)	(1.49)	(2.87)	(10.00)
Month_4	−0.96	−0.36	−4.23	0.57	0.68	−9.82***	−14.12
	(1.64)	(1.54)	(6.08)	(1.28)	(1.45)	(2.80)	(9.76)
Month_5	−4.49***	−5.77***	−24.35***	−3.20**	−5.80***	−18.54***	−62.16***
	(1.67)	(1.58)	(6.23)	(1.31)	(1.49)	(2.87)	(9.99)
Month_6	0.05	−5.01**	−19.79**	5.69***	−7.06***	−22.09***	−48.22***
	(2.19)	(2.06)	(8.14)	(1.71)	(1.94)	(3.75)	(13.06)
Month_7	1.48	0.63	−4.98	1.20	−3.69**	−8.06***	−13.42
	(1.69)	(1.60)	(6.31)	(1.32)	(1.51)	(2.91)	(10.12)
Month_8	5.29***	1.76	3.40	−2.73**	−0.59	−4.18	2.95
	(1.73)	(1.63)	(6.44)	(1.35)	(1.54)	(2.97)	(10.33)
Month_9	4.90***	3.16**	3.26	−1.74	−2.00	−4.95*	2.64
	(1.66)	(1.57)	(6.19)	(1.30)	(1.48)	(2.85)	(9.93)
Month_10	1.84	5.75***	−1.58	0.15	−2.17	0.47	4.46
	(1.66)	(1.57)	(6.19)	(1.30)	(1.48)	(2.85)	(9.93)
Month_11	1.12	5.49***	4.50	0.60	−4.99***	−4.78*	1.95
	(1.68)	(1.59)	(6.27)	(1.31)	(1.50)	(2.89)	(10.05)
Constant	10.90***	17.35***	74.58***	9.60***	24.12***	48.98***	185.54***
	(1.24)	(1.16)	(4.60)	(0.96)	(1.10)	(2.12)	(7.38)
Observations	386	386	386	386	386	386	386
Adjusted R^2	0.16	0.30	0.30	0.11	0.49	0.46	0.49

Table A2: Level of Dental Services and the Pandemic (S). The table presents the estimated slope coefficients for the number services offered in the Subsidized (S) clinic of Tehran University of Medical Sciences by each dental treatment group and the pandemic indicator variable (COVID). The regressions also include the month-fixed effects. The sample period is from April 21, 2019, to April 21, 2021 at the daily frequency. P-values are estimated using robust standard errors (reported in parentheses). ***, **, and * denote statistical significance at the 1%, 5%, and 10% p-value levels, respectively.

$$\text{Service}_t^{\text{Group}_j,S} = \alpha + \beta^{\text{Group}_j}\ \text{COVID}_t + \gamma^m\ \text{Month}_t^m + e_t^{\text{Group}_j,S}$$

	Prosthodontics	Periodontics	Pediatric	Orthodontic	Endodontic	Restorative	All Groups
COVID	−2.29***	0.67	−14.34***	2.33***	−1.64***	−2.29***	−17.55***
	(0.79)	(0.62)	(1.75)	(0.47)	(0.37)	(0.71)	(2.98)
Month_3	0.84	1.38	9.47***	1.84*	0.76	2.88**	17.16***
	(1.56)	(1.23)	(3.47)	(0.94)	(0.73)	(1.40)	(5.91)
Month_4	−1.95	−1.19	−4.35	0.34	−2.58***	−3.13**	−12.87**
	(1.49)	(1.18)	(3.33)	(0.90)	(0.70)	(1.34)	(5.66)
Month_5	−0.24	−1.05	9.82**	−2.37**	−1.60*	0.60	5.16
	(1.93)	(1.53)	(4.31)	(1.16)	(0.91)	(1.74)	(7.34)
Month_6	1.56	−1.50	3.64	9.57***	−0.05	−2.07	11.15
	(2.00)	(1.58)	(4.46)	(1.21)	(0.94)	(1.80)	(7.59)
Month_7	1.14	3.09**	7.53**	3.54***	−0.34	0.87	15.83***
	(1.58)	(1.25)	(3.52)	(0.95)	(0.74)	(1.42)	(6.00)
Month_8	5.88***	2.81**	11.37***	−0.05	−0.59	−0.85	18.57***
	(1.58)	(1.25)	(3.52)	(0.95)	(0.74)	(1.42)	(5.99)
Month_9	3.66**	4.02***	12.52***	−0.23	−1.09	−1.01	17.86***
	(1.52)	(1.20)	(3.38)	(0.91)	(0.71)	(1.37)	(5.76)
Month_10	0.34	5.20***	8.13**	1.59*	−1.70**	2.39*	15.94***
	(1.52)	(1.20)	(3.38)	(0.91)	(0.71)	(1.37)	(5.76)
Month_11	1.48	5.82***	5.42	1.93**	−1.61**	1.21	14.26**
	(1.59)	(1.26)	(3.55)	(0.96)	(0.75)	(1.44)	(6.05)
Constant	7.23***	6.82***	32.97***	0.94	6.68***	11.64***	66.28***
	(1.14)	(0.90)	(2.53)	(0.68)	(0.53)	(1.02)	(4.31)
Observations	354	354	354	354	354	354	354
Adjusted R^2	0.09	0.15	0.23	0.28	0.11	0.09	0.20

Table A3: Level of Dental Services and the Pandemic (P). The table presents the estimated slope coefficients for the number services offered in the Private (P) clinic of Tehran University of Medical Sciences by each dental treatment group and the pandemic indicator variable (COVID). The regressions also include the month-fixed effects. The sample period is from April 21, 2019, to April 21, 2021 at the daily frequency. P-values are estimated using robust standard errors (reported in parentheses). ***, **, and * denote statistical significance at the 1%, 5%, and 10% p-value levels, respectively.

$$\text{Service}_t^{\text{Group}_j,P} = \alpha + \beta^{\text{Group}_j}\ \text{COVID}_t + \gamma^m\ \text{Month}_t^m + e_t^{\text{Group}_j,P}$$

	Prosthodontics	Periodontics	Pediatric	Orthodontic	Endodontic	Restorative	All Groups
COVID	−2.84***	−7.35***	−20.63***	−3.82***	−9.54***	−16.80***	−60.97***
	(0.42)	(0.58)	(2.36)	(0.48)	(0.64)	(1.23)	(3.33)
Month_3	0.03	−0.06	−8.73*	0.80	0.01	−5.01**	−12.96**
	(0.83)	(1.13)	(4.65)	(0.94)	(1.25)	(2.42)	(6.56)
Month_4	1.23	1.20	1.90	0.55	4.20***	−6.02**	3.05
	(0.83)	(1.14)	(4.68)	(0.95)	(1.26)	(2.43)	(6.59)
Month_5	−0.32	−0.61	−4.40	0.77	3.37**	−7.25**	−8.43
	(1.02)	(1.40)	(5.75)	(1.17)	(1.55)	(2.99)	(8.10)
Month_7	1.05	−1.54	−9.85**	−1.62	−2.64**	−7.29***	−21.90***
	(0.87)	(1.19)	(4.88)	(0.99)	(1.31)	(2.54)	(6.88)
Month_8	−0.70	−1.39	−9.53**	−3.02***	−0.64	−4.77*	−20.06***
	(0.86)	(1.17)	(4.81)	(0.98)	(1.29)	(2.50)	(6.77)
Month_9	1.15	−1.19	−10.76**	−1.87**	−1.56	−5.40**	−19.63***
	(0.82)	(1.12)	(4.62)	(0.94)	(1.24)	(2.40)	(6.52)
Month_10	1.39*	0.21	−11.27**	−1.77*	−1.10	−3.37	−15.92**
	(0.82)	(1.12)	(4.62)	(0.94)	(1.24)	(2.40)	(6.51)
Month_11	0.62	0.91	2.32	−1.30	−3.39***	−5.83**	−6.68
	(0.83)	(1.14)	(4.68)	(0.95)	(1.26)	(2.43)	(6.59)
Constant	4.12***	11.04***	44.40***	8.51***	17.63***	38.53***	124.24***
	(0.62)	(0.85)	(3.49)	(0.71)	(0.94)	(1.81)	(4.92)
Observations	336	336	336	336	336	336	336
Adjusted R^2	0.13	0.34	0.23	0.22	0.48	0.37	0.53

Table A4: Level of Dental Services and the Pandemic (S&P). The table presents the estimated slope coefficients for the number services offered in Dental School clinics of Tehran University of Medical Sciences by each dental treatment group and the pandemic indicator variable (COVID). PRIVATE is an indicator variable that takes the value of one for the services offered by the Private clinic. The regressions also include the month-fixed effects. The sample period is from April 21, 2019, to April 21, 2021 at the daily frequency. The analysis includes the daily observations when both clinics and dental treatments are offering services. P-values are estimated using robust standard errors (reported in parentheses). ***, **, and * denote statistical significance at the 1%, 5%, and 10% p-value levels, respectively.

$$\text{Service}_t^{\text{Group}_j,\text{Clinic}_i} = \alpha + \beta_1^{\text{Group}_j}\ \text{COVID}_t + \beta_2^{\text{Group}_j}\ \text{PRIVATE} + \beta_3^{\text{Group}_j}\ \text{COVID}_t \times \text{PRIVATE}^{\text{Clinic}_i} + \gamma^m\ \text{Month}_t^m + e_t^{\text{Group}_j,\text{Clinic}_i}$$

	Prosthodontics	Periodontics	Pediatric	Orthodontic	Endodontic	Restorative	All Groups
COVID	−2.45***	0.65	−14.67***	2.13***	−1.75***	−2.44**	−18.52***
	(0.66)	(0.63)	(2.23)	(0.41)	(0.53)	(1.02)	(3.39)
PRIVATE	−3.84***	1.38**	0.05	5.57***	11.40***	21.94***	36.49***
	(0.66)	(0.63)	(2.20)	(0.41)	(0.52)	(1.01)	(3.36)
COVID:PRIVATE	−0.40	−7.86***	−6.08*	−6.01***	−7.68***	−13.93***	−41.95***
	(0.94)	(0.89)	(3.14)	(0.58)	(0.75)	(1.44)	(4.78)
Month_3	0.46	0.74	0.94	1.16**	0.71	−0.76	3.26
	(0.93)	(0.88)	(3.10)	(0.58)	(0.74)	(1.43)	(4.72)
Month_4	−0.72	0.02	−1.49	0.45	0.76	−4.96***	−5.94
	(0.91)	(0.87)	(3.06)	(0.57)	(0.73)	(1.41)	(4.66)
Month_7	1.19	0.54	−0.56	0.58	−1.46*	−2.87*	−2.56
	(0.98)	(0.93)	(3.28)	(0.61)	(0.78)	(1.51)	(4.99)
Month_8	2.58***	0.65	0.89	−1.58***	−0.56	−2.95**	−0.97
	(0.94)	(0.89)	(3.14)	(0.59)	(0.75)	(1.45)	(4.79)
Month_9	2.39***	1.36	0.86	−1.10*	−1.27*	−3.35**	−1.11
	(0.90)	(0.86)	(3.02)	(0.56)	(0.72)	(1.39)	(4.61)
Month_10	0.85	2.65***	−1.60	−0.14	−1.35*	−0.63	−0.22
	(0.90)	(0.86)	(3.02)	(0.56)	(0.72)	(1.39)	(4.60)
Month_11	1.05	3.16***	5.31*	0.47	−2.31***	−2.49*	5.19
	(0.95)	(0.90)	(3.17)	(0.59)	(0.75)	(1.46)	(4.83)
Constant	7.65***	8.27***	38.80***	2.05***	6.40***	14.19***	77.36***
	(0.76)	(0.72)	(2.54)	(0.47)	(0.60)	(1.17)	(3.87)
Observations	608	608	608	608	608	608	608
Adjusted R^2	0.16	0.22	0.17	0.26	0.56	0.54	0.39

Table A5: Level of Dental Services and the Pandemic (DID test). The table presents the estimated slope coefficients for the number services offered in Dental School clinics of Tehran University of Medical Sciences by each dental treatment group and the pandemic indicator variable (COVID). PRIVATE is an indicator variable that takes the value of one for the services offered by the Private clinic. The regressions also include the month-fixed effects. The sample period is from April 21, 2019, to April 21, 2021 at the daily frequency. The analysis includes all daily observations. P-values are estimated using robust standard errors (reported in parentheses). ***, **, and * denote statistical significance at the 1%, 5%, and 10% p-value levels, respectively.

$$\text{Service}_t^{\text{Group}_j,\text{Clinic}_i} = \alpha + \beta_1^{\text{Group}_j}\ \text{COVID}_t + \beta_2^{\text{Group}_j}\ \text{PRIVATE} + \beta_3^{\text{Group}_j}\ \text{COVID}_t \times \text{PRIVATE}^{\text{Clinic}_i} + \gamma^m\ \text{Month}_t^m + e_t^{\text{Group}_j,\text{Clinic}_i}$$

	Prosthodontics	Periodontics	Pediatric	Orthodontic	Endodontic	Restorative	All Groups
COVID	−1.44***	0.44	−8.99***	1.29***	−1.25**	−1.33	−5.64
	(0.48)	(0.55)	(1.92)	(0.39)	(0.55)	(1.08)	(3.79)
PRIVATE	−2.38***	1.21**	0.19	3.82***	7.65***	14.43***	30.48***
	(0.48)	(0.55)	(1.92)	(0.39)	(0.55)	(1.08)	(3.79)
COVID:PRIVATE	−0.49	−5.42***	−4.81*	−4.04***	−5.32***	−9.79***	−37.31***
	(0.69)	(0.78)	(2.75)	(0.55)	(0.80)	(1.55)	(5.47)
Month_3	−0.02	0.03	−1.35	0.74	−0.15	−1.38	−2.13
	(0.69)	(0.77)	(2.72)	(0.55)	(0.79)	(1.53)	(5.63)
Month_4	−0.19	0.14	−0.52	0.42	0.64	−2.85*	−2.51
	(0.69)	(0.77)	(2.72)	(0.55)	(0.79)	(1.53)	(5.63)
Month_5	−0.29	−0.60	1.51	−0.43	0.67	−2.07	−17.80***
	(0.86)	(0.97)	(3.40)	(0.69)	(0.98)	(1.91)	(6.13)
Month_6	0.21	−2.34*	−3.69	4.85***	−0.20	−3.77	−6.85
	(1.12)	(1.27)	(4.45)	(0.90)	(1.29)	(2.50)	(9.20)
Month_7	0.38	0.04	−2.27	0.29	−1.39*	−3.07**	−6.03
	(0.69)	(0.78)	(2.74)	(0.55)	(0.79)	(1.54)	(5.67)
Month_8	1.22*	−0.07	−1.50	−1.16**	−0.98	−2.93*	−5.42
	(0.69)	(0.78)	(2.74)	(0.55)	(0.79)	(1.54)	(5.67)
Month_9	1.74**	1.16	1.27	−0.55	−0.69	−1.61	1.31
	(0.69)	(0.78)	(2.74)	(0.55)	(0.79)	(1.54)	(5.67)
Month_10	0.71	2.12***	−0.16	0.13	−0.65	0.45	2.60
	(0.69)	(0.78)	(2.74)	(0.55)	(0.79)	(1.54)	(5.67)
Month_11	0.29	1.70**	0.93	0.14	−1.86**	−1.96	−0.76
	(0.69)	(0.78)	(2.74)	(0.55)	(0.79)	(1.54)	(5.67)
Constant	5.01***	5.38***	25.62***	1.31***	4.36***	9.32***	47.26***
	(0.57)	(0.64)	(2.26)	(0.46)	(0.65)	(1.27)	(4.61)
Observations	1,065	1,065	1,065	1,064	1,064	1,065	1,096
Adjusted R^2	0.07	0.09	0.06	0.12	0.25	0.22	0.12

Table A6: Change in Dental Services and the Pandemic (S+P). The table presents the estimated slope coefficients for the percentage changes in the number of services in the post-COVID period offered in Dental School clinics of Tehran University of Medical Sciences by each dental treatment group and the pandemic indicator variable (COVID). The growth rate of services for the observations in each month is measured by the average values in the same month of the year in the pre-COVID period. The regressions also include the month-fixed effects. The sample period is from April 21, 2019, to April 21, 2021 at the daily frequency. P-values are estimated using robust standard errors (reported in parentheses). ***, **, and * denote statistical significance at the 1%, 5%, and 10% p-value levels, respectively.

$$\frac{\text{Service}_t^{\text{Group}_j}}{avg\left(\text{Service}_{m,preCOVID}^{\text{Group}_j}\right)} - 1 = \alpha + \beta^{\text{Group}_j}\ \text{COVID}_t + \gamma^m\ \text{Month}_t^m + e_t^{\text{Group}_j}$$

	Prosthodontics	Periodontics	Pediatric	Orthodontic	Endodontic	Restorative	All Groups
COVID	−0.23***	−0.27***	−0.43***	−0.07	−0.48***	−0.40***	−0.41***
	(0.08)	(0.04)	(0.05)	(0.07)	(0.03)	(0.03)	(0.03)
Month_3	0.48***	0.33***	0.02	0.06	0.07	0.03	0.05
	(0.15)	(0.08)	(0.09)	(0.13)	(0.07)	(0.07)	(0.06)
Month_4	0.22	0.29***	0.16*	−0.06	0.15**	0.08	0.15***
	(0.15)	(0.08)	(0.09)	(0.13)	(0.07)	(0.07)	(0.06)
Month_7	0.11	0.10	−0.05	0.22	−0.02	−0.15**	−0.07
	(0.16)	(0.09)	(0.09)	(0.14)	(0.07)	(0.07)	(0.06)
Month_8	0.09	0.07	−0.04	−0.02	0.12*	−0.02	0.005
	(0.16)	(0.08)	(0.09)	(0.14)	(0.07)	(0.07)	(0.06)
Month_9	0.01	0.05	−0.03	−0.15	0.03	−0.06	−0.02
	(0.14)	(0.08)	(0.09)	(0.14)	(0.07)	(0.07)	(0.06)
Month_10	0.27*	0.26***	−0.03	0.21	0.13**	0.15**	0.10*
	(0.15)	(0.08)	(0.09)	(0.13)	(0.07)	(0.07)	(0.06)
Month_11	0.43***	0.28***	0.11	0.52***	0.19***	0.12*	0.19***
	(0.15)	(0.08)	(0.09)	(0.14)	(0.07)	(0.07)	(0.06)
Constant	−0.19*	−0.17***	−0.02	−0.10	−0.08	−0.02	−0.05
	(0.11)	(0.06)	(0.07)	(0.11)	(0.05)	(0.05)	(0.04)
Observations	300	315	321	300	323	323	327
Adjusted R^2	0.07	0.17	0.22	0.09	0.38	0.34	0.41

Table A7: Change in Dental Services and the Pandemic (S). The table presents the estimated slope coefficients for the percentage changes in the number of services in the post-COVID period offered in the Subsidized (S) clinic of Tehran University of Medical Sciences by each dental treatment group and the pandemic indicator variable (COVID). The growth rate of services for the observations in each month is measured by the average values in the same month of the year in the pre-COVID period. The regressions also include the month-fixed effects. The sample period is from April 21, 2019, to April 21, 2021 at the daily frequency. P-values are estimated using robust standard errors (reported in parentheses). ***, **, and * denote statistical significance at the 1%, 5%, and 10% p-value levels, respectively.

$$\frac{\text{Service}_t^{\text{Group}_j,S}}{avg\left(\text{Service}_{m,preCOVID}^{\text{Group}_j,S}\right)} - 1 = \alpha + \beta^{\text{Group}_j}\ \text{COVID}_t + \gamma^m\ \text{Month}_t^m + e_t^{\text{Group}_j,S}$$

	Prosthodontics	Periodontics	Pediatric	Orthodontic	Endodontic	Restorative	All Groups
COVID	−0.06	0.27***	−0.29***	0.87***	−0.09	−0.01	−0.12**
	(0.11)	(0.08)	(0.05)	(0.16)	(0.08)	(0.07)	(0.05)
Month_3	0.76***	0.66***	0.07	−0.03	0.06	0.08	0.20**
	(0.21)	(0.16)	(0.10)	(0.30)	(0.15)	(0.13)	(0.09)
Month_4	0.37*	1.15***	0.50***	0.16	1.16***	0.76***	0.72***
	(0.21)	(0.16)	(0.10)	(0.31)	(0.15)	(0.13)	(0.09)
Month_7	0.21	0.24	−0.0003	1.31***	0.15	0.10	0.11
	(0.21)	(0.17)	(0.11)	(0.31)	(0.16)	(0.14)	(0.10)
Month_8	0.10	0.16	0.06	−0.10	0.09	0.05	0.12
	(0.21)	(0.16)	(0.11)	(0.33)	(0.16)	(0.13)	(0.10)
Month_9	0.02	0.03	0.02	−0.30	−0.03	0.02	0.04
	(0.20)	(0.16)	(0.10)	(0.31)	(0.15)	(0.13)	(0.09)
Month_10	0.20	0.36**	0.01	0.45	0.17	0.54***	0.23**
	(0.20)	(0.16)	(0.10)	(0.30)	(0.16)	(0.13)	(0.09)
Month_11	0.29	0.22	0.17	0.78**	0.05	0.57***	0.35***
	(0.22)	(0.17)	(0.11)	(0.31)	(0.16)	(0.14)	(0.10)
Constant	−0.23	−0.34***	−0.10	−0.32	−0.21*	−0.26***	−0.22***
	(0.15)	(0.12)	(0.08)	(0.22)	(0.11)	(0.10)	(0.07)
Observations	272	301	316	234	291	301	316
Adjusted R^2	0.04	0.23	0.17	0.20	0.23	0.19	0.22

Table A8: Change in Dental Services and the Pandemic (P). The table presents the estimated slope coefficients for the percentage changes in the number of services in the post-COVID period offered in the Private clinic of Tehran University of Medical Sciences by each dental treatment group and the pandemic indicator variable (COVID). The growth rate of services for the observations in each month is measured by the average values in the same month of the year in the pre-COVID period. The regressions also include the month-fixed effects. The sample period is from April 21, 2019, to April 21, 2021 at the daily frequency. P-values are estimated using robust standard errors (reported in parentheses). ***, **, and * denote statistical significance at the 1%, 5%, and 10% p-value levels, respectively.

$$\frac{\text{Service}_t^{\text{Group}_j,P}}{avg\left(\text{Service}_{m,preCOVID}^{\text{Group}_j,P}\right)} - 1 = \alpha + \beta^{\text{Group}_j}\ \text{COVID}_t + \gamma^m\ \text{Month}_t^m + e_t^{\text{Group}_j,P}$$

	Prosthodontics	Periodontics	Pediatric	Orthodontic	Endodontic	Restorative	All Groups
COVID	−0.37***	−0.55***	−0.39***	−0.30***	−0.54***	−0.46***	−0.54***
	(0.09)	(0.06)	(0.07)	(0.06)	(0.03)	(0.04)	(0.03)
Month_3	0.02	0.08	0.07	0.01	0.05	−0.01	−0.02
	(0.17)	(0.10)	(0.13)	(0.11)	(0.07)	(0.07)	(0.06)
Month_4	0.18	0.12	0.05	−0.13	0.10	−0.02	0.02
	(0.18)	(0.11)	(0.12)	(0.11)	(0.07)	(0.07)	(0.06)
Month_7	0.05	0.04	0.08	−0.07	0.02	−0.13*	−0.14**
	(0.20)	(0.13)	(0.14)	(0.12)	(0.07)	(0.08)	(0.06)
Month_8	0.05	0.01	0.07	−0.02	0.11*	−0.08	−0.09
	(0.20)	(0.12)	(0.14)	(0.13)	(0.07)	(0.07)	(0.06)
Month_9	0.01	−0.01	−0.05	−0.13	0.03	−0.10	−0.08
	(0.16)	(0.11)	(0.12)	(0.12)	(0.07)	(0.07)	(0.06)
Month_10	0.05	0.05	−0.07	0.03	0.10	0.01	0.002
	(0.16)	(0.10)	(0.12)	(0.12)	(0.07)	(0.07)	(0.06)
Month_11	0.04	0.02	−0.002	0.21*	0.12*	−0.04	0.03
	(0.17)	(0.10)	(0.12)	(0.11)	(0.07)	(0.07)	(0.06)
Constant	−0.05	−0.04	−0.02	0.01	−0.06	0.04	0.03
	(0.13)	(0.08)	(0.09)	(0.08)	(0.05)	(0.05)	(0.04)
Observations	211	262	278	264	305	304	315
Adjusted R^2	0.06	0.26	0.11	0.09	0.44	0.35	0.53

Table A9: Change in Dental Services and the Pandemic (S&P). The table presents the estimated slope coefficients for the percentage changes in the number of services in the post-COVID period offered in Dental School clinics of Tehran University of Medical Sciences by each dental treatment group and the pandemic indicator variable (COVID). PRIVATE is an indicator variable that takes the value of one for the services offered by the Private clinic. The growth rate of services for the observations in each month is measured by the average values in the same month of the year in the pre-COVID period. The regressions also include the month-fixed effects. The sample period is from April 21, 2019, to April 21, 2021 at the daily frequency. The analysis includes the daily observations when both clinics and dental treatments are offering services. P-values are estimated using robust standard errors (reported in parentheses). ***, **, and * denote statistical significance at the 1%, 5%, and 10% p-value levels, respectively.

$$\frac{\text{Service}_t^{\text{Group}_j,\text{Clinic}_i}}{avg\left(\text{Service}_{m,preCOVID}^{\text{Group}_j,\text{Clinic}_i}\right)} - 1 = \alpha + \beta_1^{\text{Group}_j}\ \text{COVID}_t + \beta_2^{\text{Group}_j}\ \text{PRIVATE} + \beta_3^{\text{Group}_j}\ \text{COVID}_t \times \text{PRIVATE}^{\text{Clinic}_i} + \gamma^m\ \text{Month}_t^m + e_t^{\text{Group}_j,\text{Clinic}_i}$$

	Prosthodontics	Periodontics	Pediatric	Orthodontic	Endodontic	Restorative	All Groups
COVID	−0.10	0.11*	−0.35***	0.44***	−0.16***	−0.07	−0.16***
	(0.10)	(0.06)	(0.06)	(0.09)	(0.04)	(0.04)	(0.04)
PRIVATE	−0.39***	0.18***	0.03	0.81***	0.90***	0.96***	0.41***
	(0.10)	(0.06)	(0.06)	(0.08)	(0.04)	(0.04)	(0.04)
COVID:PRIVATE	−0.21	−0.71***	−0.04	−0.82***	−0.60***	−0.61***	−0.48***
	(0.15)	(0.09)	(0.08)	(0.12)	(0.06)	(0.06)	(0.05)
Month_3	0.48***	0.29***	0.07	0.003	0.06	0.02	0.07
	(0.15)	(0.08)	(0.08)	(0.11)	(0.06)	(0.06)	(0.05)
Month_4	0.29*	0.27***	0.18**	−0.22**	0.12**	0.07	0.17***
	(0.15)	(0.08)	(0.08)	(0.11)	(0.06)	(0.06)	(0.05)
Month_7	0.16	0.15	0.03	0.33***	0.07	−0.06	−0.03
	(0.16)	(0.09)	(0.09)	(0.12)	(0.06)	(0.06)	(0.05)
Month_8	0.17	0.09	0.07	−0.10	0.10*	−0.04	0.002
	(0.16)	(0.09)	(0.09)	(0.12)	(0.06)	(0.06)	(0.05)
Month_9	0.06	0.04	−0.01	−0.19*	0.01	−0.07	−0.02
	(0.14)	(0.08)	(0.08)	(0.11)	(0.06)	(0.06)	(0.05)
Month_10	0.19	0.22***	−0.03	0.05	0.08	0.14**	0.10*
	(0.14)	(0.08)	(0.08)	(0.11)	(0.06)	(0.06)	(0.05)
Month_11	0.29*	0.14	0.06	0.27**	0.04	0.02	0.12**
	(0.15)	(0.09)	(0.08)	(0.11)	(0.06)	(0.06)	(0.05)
Constant	−0.01	−0.24***	−0.06	−0.51***	−0.53***	−0.50***	−0.26***
	(0.12)	(0.07)	(0.07)	(0.09)	(0.05)	(0.05)	(0.04)
Observations	484	560	594	503	596	608	631
Adjusted R^2	0.09	0.16	0.11	0.20	0.57	0.55	0.39

Table A10: Change in Dental Services and the Pandemic (DID test). The table presents the estimated slope coefficients for the percentage changes in the number of services in the post-COVID period offered in Dental School clinics of Tehran University of Medical Sciences by each dental treatment group and the pandemic indicator variable (COVID). PRIVATE is an indicator variable that takes the value of one for the services offered by the Private clinic. The growth rate of services for the observations in each month is measured by the average values in the same month of the year in the pre-COVID period. The regressions also include the month-fixed effects. The sample period is from April 21, 2019, to April 21, 2021 at the daily frequency. The analysis includes all daily observations. P-values are estimated using robust standard errors (reported in parentheses). ***, **, and * denote statistical significance at the 1%, 5%, and 10% p-value levels, respectively.

$$\frac{\text{Service}_t^{\text{Group}_j,\text{Clinic}_i}}{avg\left(\text{Service}_{m,preCOVID}^{\text{Group}_j,\text{Clinic}_i}\right)} - 1 = \alpha + \beta_1^{\text{Group}_j}\ \text{COVID}_t + \beta_2^{\text{Group}_j}\ \text{PRIVATE} + \beta_3^{\text{Group}_j}\ \text{COVID}_t \times \text{PRIVATE}^{\text{Clinic}_i} + \gamma^m\ \text{Month}_t^m + e_t^{\text{Group}_j,\text{Clinic}_i}$$

	Prosthodontics	Periodontics	Pediatric	Orthodontic	Endodontic	Restorative	All Groups
COVID	−0.01	0.13***	−0.13***	0.29***	−0.04	0.002	−0.05***
	(0.03)	(0.03)	(0.02)	(0.04)	(0.03)	(0.02)	(0.02)
PRIVATE	0.00	0.00	−0.00	−0.0001	0.001	0.00	0.00
	(0.03)	(0.03)	(0.02)	(0.04)	(0.02)	(0.02)	(0.02)
PRIVATE	−0.08*	−0.30***	−0.01	−0.38***	−0.19***	−0.19***	−0.19***
	(0.04)	(0.04)	(0.03)	(0.06)	(0.04)	(0.03)	(0.03)
Month_2	−0.08	−0.10**	−0.12***	0.002	−0.14***	−0.11***	−0.14***
	(0.05)	(0.05)	(0.04)	(0.07)	(0.04)	(0.04)	(0.03)
Month_3	0.04	0.04	−0.08**	0.01	−0.13***	−0.10***	−0.11***
	(0.05)	(0.05)	(0.04)	(0.07)	(0.04)	(0.04)	(0.03)
Month_4	0.03	0.27***	0.04	0.03	0.23***	0.11***	0.09***
	(0.06)	(0.05)	(0.04)	(0.07)	(0.05)	(0.04)	(0.03)
Month_5	−0.00	0.00	0.00	0.00	−0.00	0.00	0.00
	(0.06)	(0.05)	(0.04)	(0.07)	(0.05)	(0.04)	(0.03)
Month_6	−0.00	0.00	0.00	−0.00	0.00	−0.00	−0.00
	(0.06)	(0.05)	(0.04)	(0.07)	(0.05)	(0.04)	(0.03)
Month_7	−0.01	−0.0002	−0.07*	0.28***	−0.07	−0.10**	−0.14***
	(0.06)	(0.05)	(0.04)	(0.07)	(0.05)	(0.04)	(0.03)
Month_8	−0.04	−0.05	−0.06	0.01	−0.11**	−0.14***	−0.15***
	(0.06)	(0.05)	(0.04)	(0.07)	(0.05)	(0.04)	(0.03)
Month_9	−0.13**	−0.14***	−0.16***	−0.11	−0.21***	−0.18***	−0.20***
	(0.06)	(0.05)	(0.04)	(0.07)	(0.05)	(0.04)	(0.03)
Month_10	−0.08	−0.03	−0.18***	0.17**	−0.11**	0.04	−0.10***
	(0.06)	(0.05)	(0.04)	(0.07)	(0.05)	(0.04)	(0.03)
Month_11	−0.05	−0.08	−0.10**	0.29***	−0.13***	0.02	−0.05
	(0.06)	(0.05)	(0.04)	(0.07)	(0.05)	(0.04)	(0.03)
[illegible]	[illegible]	[illegible]	[illegible]	[illegible]	[illegible]	[illegible]	[illegible]

Table A11: Level of Relative Dental Services and the Pandemic. The table presents the estimated slope coefficients for the number of services the Subsidized clinic of Tehran University of Medical Sciences offered in excess of its Private clinic by each dental treatment group and the pandemic indicator variable (COVID). The regressions also include the month-fixed effects. The sample period is from April 21, 2019, to April 21, 2021 at the daily frequency. P-values are estimated using robust standard errors (reported in parentheses). ***, **, and * denote statistical significance at the 1%, 5%, and 10% p-value levels, respectively.

$$\text{Service}_t^{\text{Group}_j,S} - \text{Service}_t^{\text{Group}_j,S} = \alpha + \beta^{\text{Group}_j}\ \text{COVID}_t + \gamma^m\ \text{Month}_t^m + e_t^{\text{Group}_j}$$

	Prosthodontics	Periodontics	Pediatric	Orthodontic	Endodontic	Restorative	All Groups
COVID	0.51	7.09***	2.19	4.84***	7.35***	12.65***	41.68***
	(1.43)	(1.03)	(3.05)	(0.69)	(0.74)	(1.41)	(4.05)
Month_3	2.91	0.43	13.17**	0.53	0.21	6.25**	26.97***
	(2.45)	(1.80)	(5.83)	(1.17)	(1.37)	(2.70)	(7.95)
Month_4	−3.46	−2.94	−6.77	0.45	−6.61***	1.82	−17.98**
	(2.72)	(1.89)	(5.44)	(1.27)	(1.39)	(2.74)	(7.84)
Month_7	−1.40	2.85	11.66*	3.98***	0.68	3.15	32.25***
	(2.75)	(2.21)	(6.26)	(1.29)	(1.57)	(3.08)	(8.41)
Month_8	9.31***	1.69	14.24**	2.79*	0.33	2.41	38.20***
	(2.91)	(2.03)	(6.42)	(1.48)	(1.41)	(2.76)	(8.06)
Month_9	4.66*	3.82**	22.17***	1.12	0.70	3.52	37.10***
	(2.39)	(1.85)	(5.51)	(1.26)	(1.36)	(2.67)	(7.75)
Month_10	−0.40	3.72**	19.40***	2.53**	−0.24	4.97*	31.43***
	(2.35)	(1.80)	(5.41)	(1.28)	(1.38)	(2.63)	(7.75)
Month_11	3.17	4.81**	0.54	1.99	0.99	6.77**	17.40**
	(2.58)	(1.92)	(5.64)	(1.21)	(1.50)	(2.80)	(8.13)
Constant	2.05	−3.01**	−9.42**	−6.45***	−10.66***	−25.17***	−56.65***
	(1.86)	(1.36)	(4.11)	(0.86)	(1.04)	(2.01)	(5.87)
Observations	182	248	273	195	273	285	304
Adjusted R^2	0.10	0.23	0.13	0.21	0.34	0.24	0.39

Table A12: Level of Relative Dental Services and the Pandemic. The table presents the estimated slope coefficients for the number of services the Subsidized clinic of Tehran University of Medical Sciences offered in excess of its Private clinic by each dental treatment group and the pandemic indicator variable (COVID). The regressions also include the month-fixed effects. The sample period is from April 21, 2019, to April 21, 2021 at the daily frequency. P-values are estimated using robust standard errors (reported in parentheses). ***, **, and * denote statistical significance at the 1%, 5%, and 10% p-value levels, respectively.

$$\text{Service}_t^{\text{Group}_j,S} - \text{Service}_t^{\text{Group}_j,S} = \alpha + \beta^{\text{Group}_j}\,\text{COVID}_t + \gamma^m\,\text{Month}_t^m + e_t^{\text{Group}_j}$$

	Prosthodontics	Periodontics	Pediatric	Orthodontic	Endodontic	Restorative	All Groups
COVID	0.51	7.09***	2.19	4.84***	7.35***	12.65***	41.68***
	(1.43)	(1.03)	(3.05)	(0.69)	(0.74)	(1.41)	(4.05)
Month_3	2.91	0.43	13.17**	0.53	0.21	6.25**	26.97***
	(2.45)	(1.80)	(5.83)	(1.17)	(1.37)	(2.70)	(7.95)
Month_4	−3.46	−2.94	−6.77	0.45	−6.61***	1.82	−17.98**
	(2.72)	(1.89)	(5.44)	(1.27)	(1.39)	(2.74)	(7.84)
Month_7	−1.40	2.85	11.66*	3.98***	0.68	3.15	32.25***
	(2.75)	(2.21)	(6.26)	(1.29)	(1.57)	(3.08)	(8.41)
Month_8	9.31***	1.69	14.24**	2.79*	0.33	2.41	38.20***
	(2.91)	(2.03)	(6.42)	(1.48)	(1.41)	(2.76)	(8.06)
Month_9	4.66*	3.82**	22.17***	1.12	0.70	3.52	37.10***
	(2.39)	(1.85)	(5.51)	(1.26)	(1.36)	(2.67)	(7.75)
Month_10	−0.40	3.72**	19.40***	2.53**	−0.24	4.97*	31.43***
	(2.35)	(1.80)	(5.41)	(1.28)	(1.38)	(2.63)	(7.75)
Month_11	3.17	4.81**	0.54	1.99	0.99	6.77**	17.40**
	(2.58)	(1.92)	(5.64)	(1.21)	(1.50)	(2.80)	(8.13)
Constant	2.05	−3.01**	−9.42**	−6.45***	−10.66***	−25.17***	−56.65***
	(1.86)	(1.36)	(4.11)	(0.86)	(1.04)	(2.01)	(5.87)
Observations	182	248	273	195	273	285	304
Adjusted R^2	0.10	0.23	0.13	0.21	0.34	0.24	0.39
